# Spatial variation in the frequency of knockdown resistance genotypes in Florida *Aedes aegypti* populations

**DOI:** 10.1186/s13071-020-04112-3

**Published:** 2020-05-11

**Authors:** Stephanie J. Mundis, Alden S. Estep, Christy M. Waits, Sadie J. Ryan

**Affiliations:** 1 Quantitative Disease Ecology and Conservation (QDEC) Lab, Department of Geography, University of Florida, Gainesville, FL 32607 USA; 2 Emerging Pathogens Institute, University of Florida, Gainesville, FL 32608 USA; 3 Navy Entomology Center of Excellence, R&D Department, Gainesville, FL 32608 USA; 4 School of Life Sciences, University of KwaZulu-Natal, Durban, South Africa

**Keywords:** Insecticide resistance, *Aedes aegypti*, Spatial scan statistic, Beta regression, Knockdown resistance

## Abstract

**Background:**

The development of insecticide resistance in disease-vectoring mosquito species can lead to vector control failure and disease resurgence. However, insecticide applications remain an essential public health intervention. In Florida, insecticide resistance in *Aedes aegypti*, an anthropophilic mosquito species capable of transmitting dengue, chikungunya, and Zika virus, is a major concern. Understanding the location, scale, and driving factors of insecticide resistance can enhance the ability of vector control organizations to target populations effectively.

**Methods:**

We used previously collected data on frequencies of mutations that confer resistance to commonly used pyrethroid insecticides in *Ae. aegypti* specimens from 62 sites distributed across 18 counties in Florida. To determine the scale of clustering for the most resistant variant, we used a Ripley’s K function. We also used a spatial scanning statistic technique to identify locations of clusters where higher than expected frequencies of susceptible or resistant mosquitoes occurred. We then tested for associations between landscape, demographic, and insecticide-use factors using a beta regression modelling approach and evaluated the effect of spatial lag and spatial error terms on overall explanatory power of these models.

**Results:**

The scale at which maximum clustering of the most resistant variant occurs is approximately 20 kilometers. We identified statistically significant clusters of genotypes associated with resistance in several coastal cities, although some of these clusters were near significant clusters of susceptible mosquitoes, indicating selection pressures vary at the local scale. Vegetation density, distance from roads, and pyrethroid-use by vector control districts were consistently significant predictors of knockdown resistance genotype frequency in the top-performing beta regression models, although pyrethroid use surprisingly had a negatively associated with resistance. The incorporation of spatial lags resulted in improvements to the fit and explanatory power of the models, indicating an underlying diffusion process likely explains some of the spatial patterns observed.

**Conclusions:**

The genetic mutations that confer resistance to pyrethroids in *Ae. aegypti* mosquitoes in Florida exhibit spatial autocorrelation and patterns that can be partially explained by landscape and insecticide-use factors. Further work at local scales should be able to identify the mechanisms by which these variables influence selection for alleles associated with resistance.
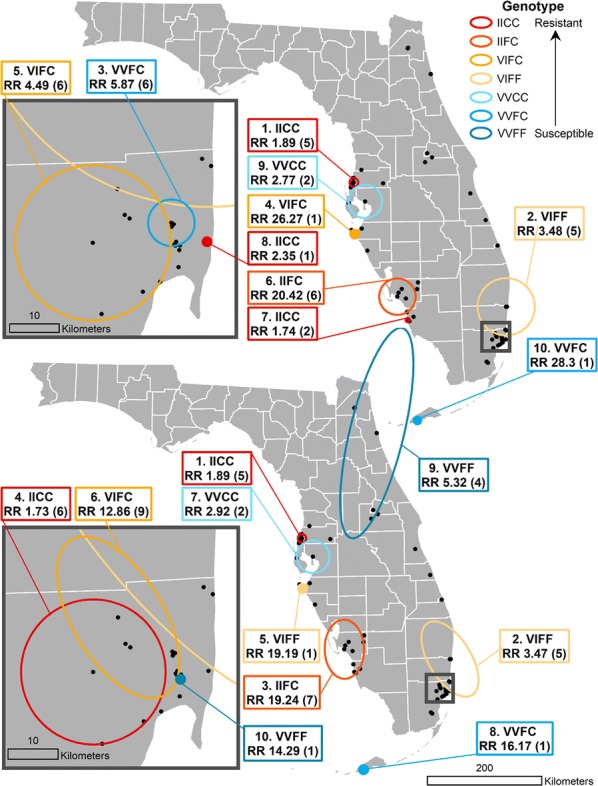

## Background

Insecticides are used globally to control mosquito populations and reduce the risk of mosquito-borne disease [1]. An unfortunate side effect of this widespread use has been the rise of insecticide resistance *via* the selection for mosquitoes that are resistant to these treatments [2, 3]. In many areas, the prevalence and intensity of insecticide resistance in mosquito vectors has increased in the last decade, leading to resurgences of mosquito-borne disease [4–6]. For diseases for which there are currently no widely available vaccines, such as Zika, chikungunya, and West Nile virus, insecticide applications are seen as the only truly effective approach for protecting the public [7]. However, when vector control organizations are not aware of the presence or extent of insecticide resistance and how resistance reduces efficacy, they may inadvertently waste time and resources applying pesticide treatments that will not reduce disease-transmitting mosquito populations.

**Fig. 1:**
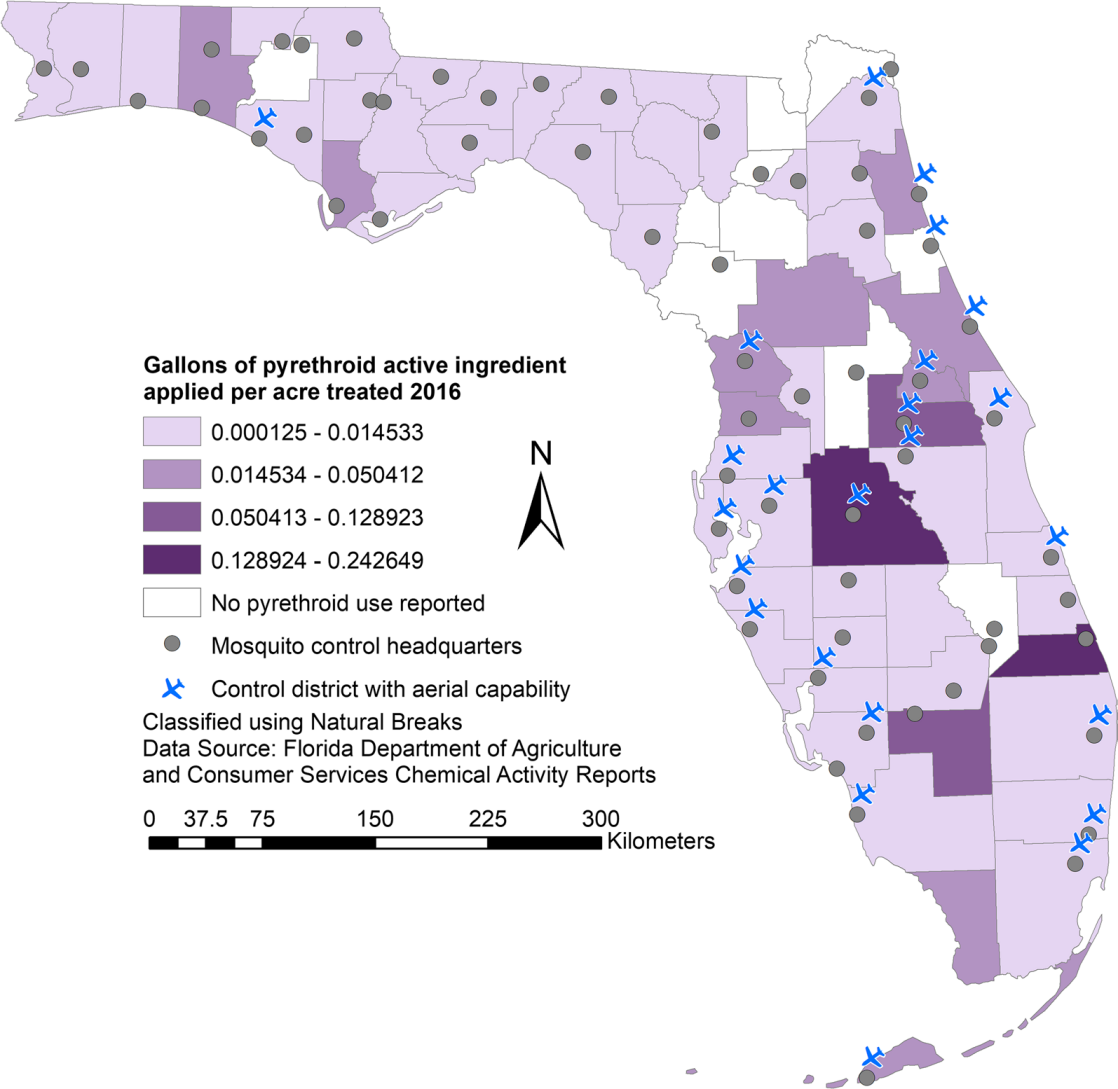
Map of pyrethroid application intensity by gallons of active ingredient per the number of acres treated by vector control districts. Headquarters of control districts and their current aerial capability are also shown. Data is based on 2016 Florida Department of Agriculture and Consumer Services Chemical Activity Reports

In Florida, insecticides are widely used to control the mosquito species that transmit disease as well as nuisance species [8]. There are currently 61 active mosquito control programmes throughout the state, most of which are organized at the county level [9] (Fig. 1), in addition to numerous private pest control companies that perform mosquito control. Vector control districts generally aim to implement integrated pest management including efforts in public education and outreach, larval breeding site reduction, and the application of biological controls, but the application of insecticides to kill adult mosquitoes remains the central component of vector control, particularly during an outbreak of mosquito-borne disease [9]. In Florida, insecticide application is typically done using ultra-low volume (ULV) spraying from ground vehicles or airplanes [8, 10]. Control districts most frequently use pyrethroids (e.g. permethrin and resmethrin) or organophosphates (e.g. malathion and naled), with pyrethroids being more frequently used than organophosphates throughout the state [9]. Residual spraying, usually with a handheld device, can be used to coat surfaces with insecticides and kill landing adult mosquitoes, though pest control companies use this technique more frequently than control districts [9]. Repeated exposure of mosquito populations to these treatments has led to the development of localized insecticide resistance in several species throughout the state [9, 11–13].

Monitoring insecticide resistance in *Aedes aegypti* has been a concern for vector control organizations internationally and in Florida. This species is widespread in Florida, particularly in urban areas, and is the primary vector of dengue, chikungunya and Zika viruses throughout most of the tropics and sub-tropics [14]. To monitor insecticide resistance in this and other species, researchers and control districts perform bioassays, in which sampled mosquitoes are exposed to diagnostic concentrations of insecticides inside a closed container and percent mortality is recorded at specified time intervals [15]. Additionally, two single nucleotide polymorphisms (SNPs) confer knockdown resistance (*kdr*) to pyrethroid insecticides in *Ae. aegypti* through changes in the sodium channel [16]. The first mutation at codon 1016 results in a change from a valine (V) to an isoleucine (I) [17], while the second mutation at codon 1534 results in a change from a phenylalanine (F) to a cysteine (C). The frequency of individuals with the homozygous mutant variant at both sites (IICC) has a strong positive correlation with pyrethroid resistance in tested populations and can be used to estimate on-the-ground resistance [18, 19]. Mosquitoes that are heterozygous at both sites typically exhibit limited resistance to pyrethroids [20].

In this study, we first described the spatial variation in *kdr* mutation frequencies in Florida *Ae. aegypti* populations by measuring scales of spatial dependency and identifying statistically significant clusters of *kdr* genotypes. This was done for two reasons. First, conducting bioassay-based resistance monitoring is costly, time-consuming, and logistically difficult, meaning most vector control districts can only regularly monitor a small number of sites. An understanding of the scale of spatial autocorrelation in resistance-causing genotypes across collection sites would allow the status of a site where monitoring is not conducted to be estimated based on neighboring sites. Secondly, identified clusters of resistant and susceptible genotypes can be used to inform operational management decisions. Areas with higher than expected frequencies of genetically resistant strains of mosquitoes would likely benefit from deploying alternative control strategies, while clusters of susceptible mosquitoes may play an important role in preventing fixation of resistance in the population [21].

Beyond describing the observed patterns in *kdr* mutations across Florida, there is a need to explain this variability. The development of insecticide resistance is shown to be related to the frequency and intensity of insecticide exposure [22]. However, in field settings, multiple landscape variables can modulate the degree to which mosquito populations are exposed to insecticide applications, resulting in differential selection pressure and resistance outcomes. For example, percent mortality from road-based ULV spraying in cage trials has been shown to be lower for *Ae. aegypti* in densely vegetated areas and locations further from the road [23]. Additionally, proximity to agricultural production, where insecticides are often applied regularly, has been associated with the development of resistance in multiple mosquito species [24, 25]. An understanding of the strength and direction of the relationships between the prevalence of insecticide-resistant mosquitoes and these factors could be beneficial in efforts to tailor insecticide applications for maximum effectiveness and sustainability.

There were three specific objectives for this study: (i) determine the spatial scale of clustering in frequencies of the resistant *kdr* genotype in Florida populations of *Ae. aegypti*; (ii) identify and map locations that represent statistically significant clusters of *kdr* genotypes; and (iii) test for significant associations between quantifiable landscape factors and frequencies of the resistant IICC genotype using a beta regression modeling framework.

## Methods

The data used in this study were collected as part of a statewide insecticide resistance monitoring effort that is described in detail in Estep et al. (2018) [18]. The frequencies of *kdr* genotypes were determined for *Ae. aegypti* strains collected as eggs or larvae at 62 sites across 18 counties, primarily in 2016 and 2017, although *Ae. aegypti* from one site (Hillsborough County) were collected in 2014. For 21 sites, specimens from collections in close proximity to each other were pooled to the geographic mean center of the contributing locations. Importantly, collections were not made within a single season, which could influence the probability of detecting resistance, since mosquito activity and resulting application intensity both tend to peak during the summer months.

We used the weighted Ripley’s K-function to characterize spatial dependency in the recorded frequencies of the resistant IICC genotype across the 62 sampled sites in Florida. The Ripley’s K-function summarizes the extent to which points are clustered or dispersed across a range of distance bands [26]. This is done by comparing the actual number of neighboring points present within a circle centered on an arbitrary point to the number of points that would be expected given a random distribution. In a weighted K-function analysis, the null hypothesis is that the pattern of weight values (in this case, the count of mosquitoes with the IICC genotype) assigned to the points is not significantly more clustered than the underlying pattern of the points. This analysis was implemented in ArcMap 10.6 with 30 distance bands of 5 km. A minimum enclosing rectangle, which is the smallest rectangle that can be generated to include all the sampling sites, was used as the study area for the analysis. This was chosen as an alternative to using the Florida boundaries, since sampling was not evenly distributed throughout the state and was not conducted at all in the Florida Panhandle. The Ripley’s edge correction formula was applied to account for points located near the edge of the study area. The confidence interval was computed based on 999 permutations of random point patterns, derived from the first implementation of the Ripley’s K-function on the unweighted sampling sites, while the plot of observed spatial dependency was based on the sampling sites, weighted by the count of mosquitoes with the IICC genotype.

We used SaTScan Version 9.4 [27] to identify and map clusters of the different *kdr* genotypes. This software detects clusters in space and time and tests identified clusters for statistical significance. We used the multinomial probability model [28], allowing detection of higher or lower than expected frequencies of each unique *kdr* genotype, and implemented both a circular and elliptical window shape to allow comparisons between the two methods [29]. The genotype frequencies, which ranged from 0.0 to 1.0 in the original dataset, were multiplied by 100 and rounded to the nearest integer, then imported as case counts. The population for each site was set to 100 individuals. After experimentation at different maximum spatial cluster sizes, 15% of the population at risk was used as the cut-off value. This maximum cluster size detected clusters at scales that approximated the county-level scale of vector control implementation, whereas larger thresholds yielded clusters that encompassed many counties, and smaller thresholds resulted in many clusters that only included a single site. We used 999 standard Monte Carlo replications to compute significance and allowed adjustment for more likely clusters.

Several landscape variables were considered as potential covariates in the model of pyrethroid-resistant IICC genotype frequency (Table 1). Products derived from the Moderate Resolution Imaging Spectroradiometer (MODIS) were downloaded to represent vegetation density. This included the MODIS Vegetation Continuous Fields products, which are generated annually based on monthly composites to represent percent tree cover and percent non-tree vegetation cover at a scale of 250 meters [30]. MODIS-derived enhanced vegetation index (EVI) and leaf area index (LAI) products were also included, representing vegetation conditions in January, April, July and October 2016. Because vector control districts frequently apply insecticides from ground vehicles in Florida, we also included spatial data representing the configuration of road networks. The 2016 TIGER/Line road files were downloaded from the United States Census Bureau [31], and Euclidean distance and road density were calculated using the dataset of only primary and secondary roads as well as the dataset of all roads. To represent agricultural land use, we downloaded the 2016 United States Department of Agriculture National Cropland Data Layer and calculated the Euclidean distance from all agricultural land cover and from each of the five most abundant crop types in Florida (oranges, sugar cane, hay, peanuts, and cotton) [32]. We also included data on land cover and percent impervious surface from the 2016 National Land Cover Database, published by the Multi-Resolution Land Characteristics Consortium [33]. Euclidean distances from urban or built-up land, forested land, and wetland were calculated for the study area. Finally, we included data on the 5-year estimates of median household income and population density at the census tract level from the United States Census Bureau 2016 American Community Survey [34].

**Table 1:** Variables considered for beta regression models and data source

Variable	Source	Date
Distance from agricultural land	National Cropland Data Layer	2016
Distance from oranges, sugar cane		
Distance from sugar production		
Distance from hay production		
Distance from peanut production		
Distance from cotton production		
Primary and secondary road density	United States Census Bureau TIGER Shapefiles	2016
Distance from primary and secondary road		
All roads density		
Distance from all roads		
Percent tree cover	MODIS Vegetation Continuous Fields	2016
Percent non-tree vegetation cover		
16-day composite of enhanced vegetation index (EVI)^a^	MODIS LP DAAC	2016
16-day composite of leaf area index (LAI)^b^		
Distance from urban or built-up land cover	National Land Cover Database	2016
Distance from forest land		
Distance from wetland		
Percent impervious surface		
Population density	United States Census Bureau American Community Survey	2016
Median household income		
Pyrethroid use by control district Organophosphate use by control district Total insecticide use by control district	Florida Department of Agriculture and Consumer Services	2016

^a^EVI 17 January 2016, 22 April 2016, 27 July 2016, 15 October 2016

^b^LAI 24 January 2016, 30 April 2016, 24 July 2016, 23 October 2016

All of the spatial data described above were projected to the Albers Conic Equal Area projection used by the Florida Geographic Data Library, clipped to the state boundary, and resampled to 250 m resolution, which was the coarsest spatial resolution of the input datasets. We extracted values from each dataset at the sampling site locations. In cases where multiple neighboring sites were pooled to a centroid, we generated a single standard deviation ellipse centered on the centroid to capture the dispersal of the contributing sites, and calculated the mean value for the pixels included within the ellipse.

In addition to landscape factors, we included information on insecticide use by vector control districts in 2016. Each vector control district in the state submits an annual Chemical Activity Report to the Florida Department of Agriculture and Consumer Services (FDACS), detailing the types and amounts of adulticides and larvicides used, as well as the number of acres treated. We summarized adulticide use by class as gallons of active ingredient used per acre treated for each district. We also calculated the combined amount of adulticides used in each district as the total number of gallons of active ingredient per acre treated.

We used a beta regression modelling framework to test for statistically significant associations between the variables described above and the frequency of the IICC genotype in collected *Ae. aegypti* specimens. Beta regression was selected because it is an appropriate model in cases where the dependent variable is a proportion ranging from 0 to 1, as was the case for the IICC genotype frequency [35]. Prior to implementing the model, the distributions of all explanatory variables were assessed for approximate normality using density plots and log-transformed as necessary, and each individual predictor was plotted against the response variable to determine if a linear relation would likely be appropriate. All explanatory variables were rescaled to have a mean of zero and a standard deviation of one, due to the wide-ranging scales of measurement used across the original, unscaled set of variables [36]. To assess for collinearity between the explanatory variables, we used variance inflation factors (VIF), calculated with the ‘vifstep’ function with a threshold of 10 using the *USDM* R package [37]. Ten variables were removed based on this threshold, and the remaining 22 variables were included in the full model, generated using the *betareg* R package [38]. All potential combinations of variables were considered using the ‘dredge’ function in the MuMIn R package, with a limit of six explanatory variables [39]. The models were ranked by AIC_c_, a variant of Akaike’s information criterion that is adjusted for small sample sizes, and the twenty best-performing models by this metric were considered.

We assessed the top ten models using standard regression diagnostics and tested the residual values from these models for spatial autocorrelation using the Moran’s I statistic, with a k-nearest neighbors weights matrix. We used three nearest neighbors instead of a distance-based weights matrix due to the underlying, clustered distribution of the sampling sites. When considering alternative weights matrices, smaller distances resulted in outlying sites being without neighbors, while larger distances resulted in most of the sampling locations being neighbors for more central sites. We considered spatial lag and spatial error models, using the *spdep* R package [40], comparing them to the original models using likelihood-ratio (LR) tests, Wald tests, AIC_c_, and pseudo-*R*^2^ values. These models are both extension of the non-spatial linear regression model. While the spatial lag model includes the average value of the dependent variable for the neighbors of a site as an additional independent variable, the spatial error model includes the model error values associated with the neighbors of the site [41].

## Results

**Fig. 2:**
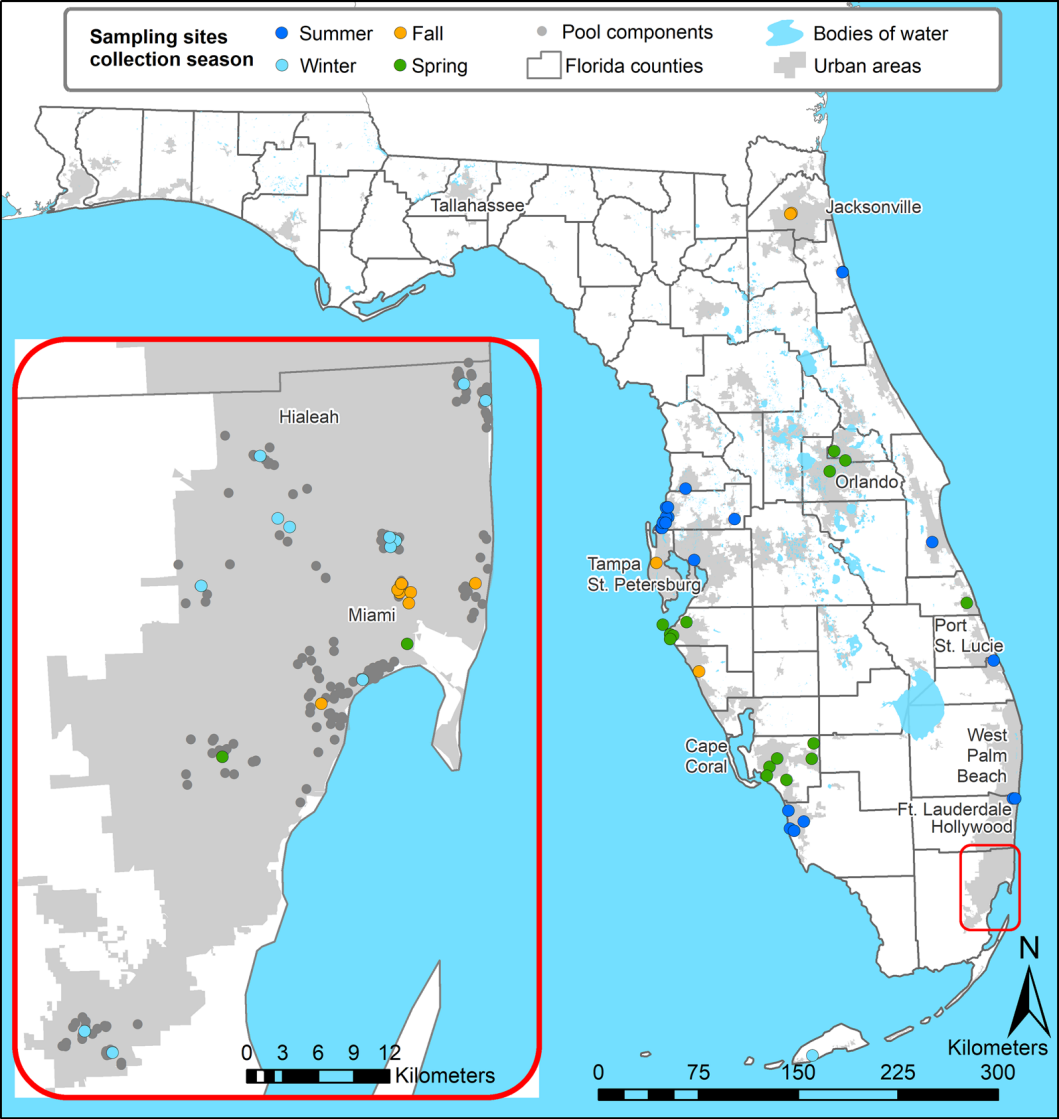
Map of sampling locations in Florida counties. Inset map shows Miami-Dade County, where more intensive sampling was conducted and neighboring sites were pooled to central locations

Collection sites and the season during which these collections were made are shown in Fig. 2 (Winter: December–February; Spring: March–May; Summer: June–August; Fall: September–November). The number of mosquitoes collected at each site ranged from 11 to 180, with an average sample size of 76 (Additional file 1: Table S1).

**Fig. 3:**
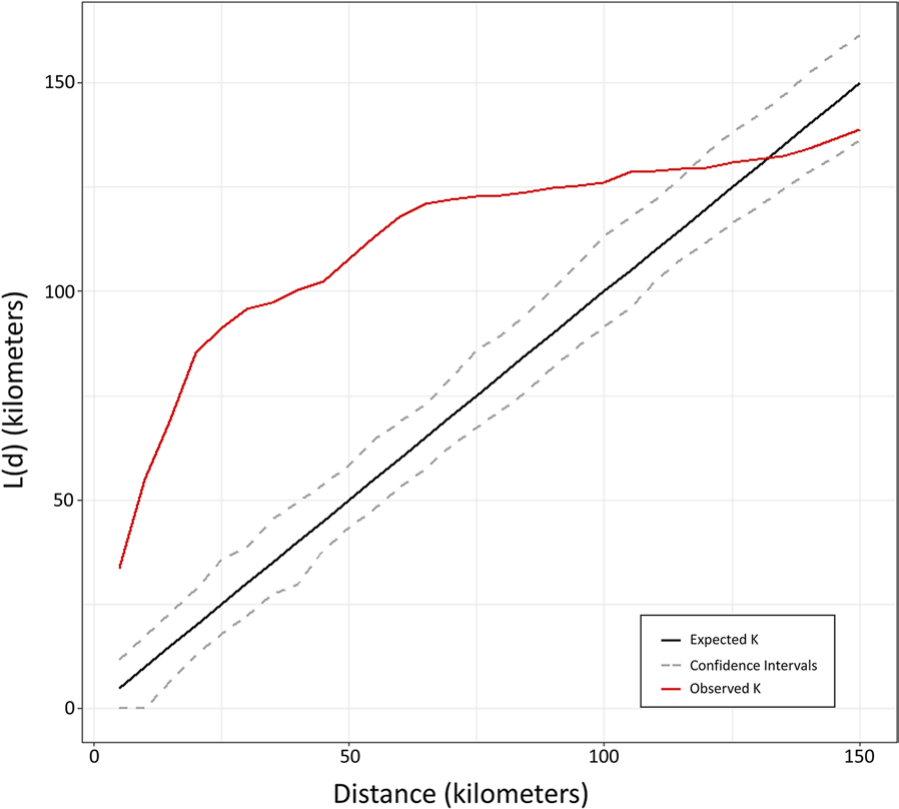
Results of Ripley’s K-function analysis. The x-axis represents the scales of clustering considered and the y-axis represents the K-function value. When the observed K has a higher value than the confidence intervals surrounding the expected K, this implies there is greater than expected clustering in the IICC genotype frequency values at that spatial scale

The results from the dual Ripley’s K analysis to detect clustering of the frequency of the IICC genotype are shown in Fig. 3. The observed K-value was furthest from the expected value at 25 km, indicating this is the scale at which maximum spatial autocorrelation in the count of *Ae. aegypti* with the IICC genotype occurs. At approximately 120 km, however, IICC count values no longer exhibit spatial dependency, indicating that sites that are more than 120 km away from each other are not more similar to each other than would be expected given a random distribution.

**Fig. 4:**
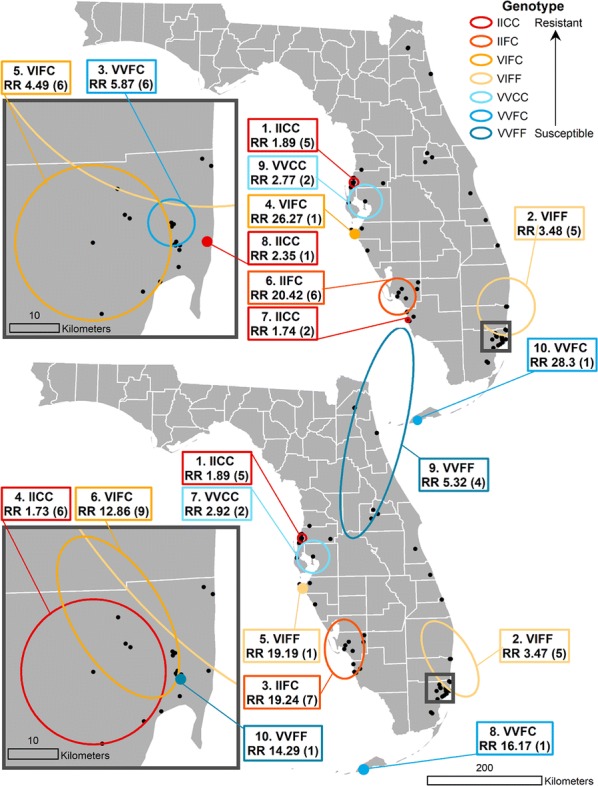
Comparison of statewide SaTScan analysis results with circular window shape (top), with inset map showing clusters identified in Miami-Dade County (top left) and elliptical window shape (bottom), with inset map showing Miami-Dade County (bottom left)

The SaTScan analyses identified clusters of higher than expected frequencies of the various *kdr* genotypes; the circular and elliptic window shapes revealed similar results, as shown in Fig. 4. Each cluster is labeled with the specific genotype that was found to have the highest relative risk (RR) of being detected in that window. Relative risk represents the estimated risk of finding that particular genotype within the identified cluster divided by the estimated risk of finding that genotype outside of the cluster [27]. Additionally, each cluster label has the number of points that were included in that cluster noted in parentheses. The first cluster detected was located on the western coast in Pasco County and consisted of five sites where high frequencies of the IICC genotype were detected, and the second cluster was located in Broward County, encapsulating five sites with high frequencies of the VIFF genotype, which is heterozygous at the 1016 locus and homozygous susceptible at the 1534 locus. Similarly, clusters of the VVCC, VIFF, and IIFC genotypes were identified along the western coast of the state in Hillsborough, Manatee and Lee County with two, one and six to seven sites (depending on circular or elliptical window shape), respectively. A cluster of the VVFC genotype consisting of a single site was detected in Monroe County, which includes the Florida Keys. Importantly, the VIFF, IIFC and IIFF genotypes are rare, meaning even low counts of these mosquitoes constituted clusters due to these genotypes not being found elsewhere in the state. There were several notable differences when comparing the clusters identified with circular *versus* elliptical window shape. In Miami-Dade County, the location and extent of the identified IICC and VIFC clusters differed depending on the window shape, while a single-site cluster of the VVFF genotype, which is the wild-type, susceptible genotype was detected with the elliptic window shape, but not the circular. Similarly, a second VVFF cluster composed of four points was detected exclusively with the elliptic window shape on the northeastern coast in Saint Johns County, extending southward to Orange County, and an IICC cluster of two sites was detected exclusively with the circular window shape in Collier County.

Twenty models were within two AIC_c_ values of the lowest AIC_c_, indicating these all had approximately equal plausibility as candidates for modelling these data [42]. All the model coefficients, AIC_c_ values and *R*^2^-values are shown in Additional file 1: Table S2. The size of the twenty candidate models ranged from four to six explanatory variables, and the *R*^2^-value ranged from 0.26 to 0.35.

In the twenty best performing models, the residuals exhibited significant spatial autocorrelation, based on the Moran’s I test with a spatial weights matrix of k = 3 nearest neighbors. The incorporation of a spatial lag and spatial error had varying effects on the AIC_c_ and *R*^2^ metrics for the candidate models (Additional file 2: Figure S1). Thirteen models had improved (lower) AIC_c_ scores with a spatial lag while seven models improved with the inclusion of a spatial error term, although only two of those models showing a decrease in AIC_c_ > 2. Additionally, the spatial lag models consistently had higher *R*^2^-values than the original or spatial error model with the same set of variables.

The best performing model, in terms of both the AIC_c_ and *R*^2^-values, was the spatial lag model with October EVI, July LAI, percent tree cover, percent non-tree vegetation, district-level pyrethroid-use, and distance from primary and secondary roads. Five additional spatial lag models were within two AIC_c_ values of this top performing model (Table 2).

**Table 2:** Summary of top-ranked spatial lag models. AIC_c_ and *R*^2^ values for six best performing spatial lag models, as well as variable coefficients and corresponding significance levels

Model	1	2	3	4	5	6
AIC_c_	− 24.82	− 24.20	− 24.17	− 23.70	− 23.68	− 23.61
*R*^2^	0.49	0.49	0.47	0.46	0.46	0.49
Intercept	0.32***	0.32***	0.30***	0.32***	0.32***	0.32
October EVI	0.05*			0.04	0.06*	
July LAI	0.07**	0.07**	0.07**	0.07**		0.08***
Percent tree cover	0.07**	0.07**		0.05*	0.07**	0.05*
Percent non-tree vegetation	− 0.05*	− 0.05			− 0.06*	
Pyrethroid use	− 0.08***	− 0.09***	− 0.08***	− 0.09***	− 0.07**	− 0.09***
Distance from roads	0.04*	0.04*	0.04	0.04	0.05*	0.08
January EVI		0.05*				0.04
Distance from forest			− 0.03			
Median income			0.07**			
January LAI					0.06**	

*Notes*: Each column corresponds with a single model. The first two rows show the AIC_c_ and *R*^2^ values for six best performing spatial lag models. The remaining rows show the variable coefficients and corresponding significance levels for each model

*P < 0.05, **P < 0.01, ****P < 0.001

## Discussion

The positive spatial autocorrelation in counts of the IICC, pyrethroid-resistant genotype at the sampled sites indicates that neighboring sites tend to have similar IR profiles for this species, particularly when sites are approximately 20 km away each other. In a scenario where the status of an *Ae. aegypti* population is unknown at one site, a vector control district could predict that it will be typically similar to neighboring sites with a known *kdr* genotype frequency. However, this estimate would merely be a starting point if data on resistance status are not immediately available and would ideally not replace an actual assessment of susceptibility. This is especially true given the limited intensive, local-scale sampling included in this dataset, which was collected with the aim of capturing statewide patterns. For example, while collections in Miami-Dade County were conducted at many sites, most of these collections were pooled to centroids. Across these pools, the average distance between sites ranged from 40 meters to 4.4 kilometers. Within more dispersed clusters, genotype frequencies may have varied across sites, but this variation at the local scale cannot be examined with this dataset because collected mosquitoes were pooled to the cluster centroid. Similarities in IICC counts decrease and approximate a random distribution at the scale of 120 km, indicating that sites from neighboring counties would not be informative in predicting IICC frequencies. For future work in estimating IICC prevalence throughout the state, interpolation methods would likely be informative. However, other studies have found reduced accuracy in multiple interpolation methods when sampling sites are highly clustered, as is the case with this dataset [43]. To address this concern and employ interpolation in this scenario, more evenly distributed sampling would need to be conducted, particularly in the central parts of the state that are not currently well represented and generally have a more rural landscape that is distinct from the well-sampled, urban coasts.

We included SaTScan analyses with both circular and elliptic scanning window shape, since the sampled points in this study were predominantly located in coastal areas, which reflects the natural distribution of *Ae. aegypti* in FL, and an elliptic shape may capture this configuration more adequately. While the results from these two analyses were similar, each approach identified at least two significant clusters that that were unique to that method. This confirms previous findings that suggest considering multiple graphical representations of clusters in SaTScan can yield clusters that would not otherwise be detected [29]. Further work in this area would benefit greatly from collecting longitudinal data on *kdr* genotype frequencies to identify the temporal variation in the identified clusters using the space-time scan statistic available in SaTScan, allowing for assessment of the stability or seasonality of the patterns identified in this study.

The areas identified as significant clusters of the IICC genotype were in coastal cities, including New Port Richey, Naples and Miami. Each of these clusters comprised four or fewer sites, indicating that these occurrences of high IICC genotype frequency may be the result of neighborhood or household-scale selective pressures. Similar work in Yucatàn State, Mexico, found significant differences in IICC frequencies between city blocks [44], and it has been shown that typical household-level insecticide use can result in selection for resistance in *Aedes aegypti* [45]. Clusters of the most susceptible genotype, VVFF, which is homozygous susceptible at both loci, were identified with centers in St. Johns County and Miami-Dade County. In the case of Miami-Dade County, this cluster could represent a localized refuge wherein mosquitoes are somehow protected from the regular applications of pyrethroid treatments, despite heavy use throughout the rest of the county. This persistence of the wild-type genotype, even if only at limited refugia, has important implications for the potential to re-establish susceptibility. Given the fitness cost of pyrethroid resistance to mosquitoes, susceptible mosquito populations would gain an advantage if the selective pressure of pyrethroid applications is removed [46].

The top-ranked beta regression models revealed a complex relationship between vegetation and insecticide resistance in *Ae. aegypti* in this system (Additional file 3: Figure S2). Based on field trials that found lower *Ae. aegypti* mortality in caged trials in areas with high vegetation density [23], we predicted lower frequencies of the IICC genotype in areas with high vegetation density. There was a negative association between percent non-tree vegetation cover and IICC genotype frequency in three of the original twenty candidate models, all of which were ultimately in the best performing spatial lag models. This indicates that areas with dense ground vegetation may shelter *Ae. aegypti* from insecticide applications and reduce the selective pressure driving insecticide resistance. However, there were significant positive associations between the variables of January or October EVI, January or July LAI, and percent tree cover, and the outcome of IICC genotype frequency. This means that areas with healthy, dense canopy cover are more likely to have pyrethroid-resistant *Ae. aegypti* populations. This could be due to overall higher abundance of multiple mosquito species in shaded, sheltered areas [47, 48], leading to a response of more liberal applications of insecticides by control districts, pest control companies, and private landowners, and overall intense selection pressure. Understanding the exact nature of the impact of vegetation on insecticide application efficacy and the development of resistance will likely require experimental field trials or fine-scale sampling across several landscape configurations.

The negative association between the intensity of pyrethroid use by control district and the frequency of the IICC genotype was unexpected. This outcome could arise due to limitations in the dataset on insecticide use, which was derived from Chemical Activity Reports submitted by mosquito control districts. In these reports, figures on the total amount of each product used and the number of acres treated were reported, but there was no delineation of the areas within each district that were treated. This means that collection sites used in this study could potentially be outside of the areas that were treated regularly and intensively with pyrethroids. Additionally, districts that have enough financial resources to purchase and apply large amounts of pyrethroids may also be more likely to have the ability to incorporate larvicides, biological control, or public education efforts into their integrated pest management plans, thus mitigating the selective pressure of pyrethroid use in some areas of the district. Finally, this dataset only included reports from mosquito control districts, while insecticide applications in this and other states are frequently conducted by private pest control companies or landowners [49]. Despite these limitations, these data represented the most accurate and current representation of insecticide use in the state, meaning it would be inadvisable to exclude them from consideration for these models. Further modelling of insecticide resistance would benefit from more detailed information on the locations and timing of insecticide treatments.

The results from the original beta regression models identified statistically significant relationships between landscape factors and the outcome of IICC frequency. However, these models only explained a portion (on average, 32%) of the variation in this response variable. The spatial lag models included information on neighboring IICC frequencies, as well as the original landscape factors, to explain variation in the response variable [41]. The spatial lag models improved the overall fit and explained a greater amount of the variation present in IICC frequencies than the non-spatial beta regression models, with a maximum *R*^2^-value of 0.49. In the spatial lag models, the coefficients of the original variables decreased, and the *P*-values associated with them increased, with some of the associations no longer being statistically significant. This indicates that including IICC frequencies of neighboring sites can dramatically improve our ability to estimate the IICC frequency at a given location and may provide more information than the landscape and insecticide-use variables originally considered. The significant improvement in the models observed with the addition of the spatial lag also indicates that there is likely a diffusion process occurring, meaning immigration between neighboring sites may explain some of the observed spatial dependence [41]. While knowledge of this spatial dependence is useful for predicting IICC frequencies if information on nearby sites is available, the original models indicate that information on landscape factors and insecticide use can explain some of the underlying variation present.

Ultimately, the best performing model explained roughly half of the variation in the frequencies of the IICC genotype. Several factors likely contribute to the remainder of that variation. Data on the history insecticide applications and resistance status throughout the county would likely improve these models. For example, if the IICC genotype had reached fixation in a population due to regular applications of pyrethroids in past years, susceptible variants would then only arise due to gene flow, which may not occur if the population is geographically isolated from susceptible strains. Additionally, information on the intensity of insecticide use by individuals and private companies would likely be informative. In a recent study across three regions in North Carolina, researchers found that approximately 31% of survey respondents applied pesticides to control mosquitoes on their properties [50]. If Florida residents are applying insecticides at a similar rate, this likely constitutes a selective pressure. To further improve the fit of these models, it would be valuable to restrict sampling to a more limited time period, since variation in IICC frequency has been shown to fluctuate seasonally [51], and include more sampling sites, which would allow the consideration of potential interactions between the explanatory variables.

This study is a demonstration of the potential for spatial analysis methods to explain and estimate frequencies of *kdr* frequencies in *Ae. aegypti* populations, with important implications for vector control. While *kdr* frequencies and the phenotype of pyrethroid resistance have been shown to vary spatially and temporally [44, 51, 52], intensive, constant monitoring of resistance is not logistically feasible. This leaves a need for tools to fill in gaps in knowledge both in time and space, although this project only focused on space. Important takeaway points from the perspective of a vector control organization include the following. First, populations within 20 kilometers of each other are likely to have similar IICC frequencies, particularly if these sites have similar landscape features. This means that if resistance is detected at one site, neighboring locations should be assumed to have similar measures, and alternative control strategies should be deployed. A second important point is that some areas in Florida have higher than expected frequencies of the wild-type genotype, meaning these populations are still susceptible to pyrethroids. These control districts may have developed programmes that curtail the development of resistance. This highlights the importance of sharing information between control districts on integrated pest management practices that are effective, sustainable, and culturally acceptable within the context of Florida. Finally, we found that pyrethroid use by control districts was negatively associated with the outcome of high IICC frequencies. While there were limitations to this dataset, as discussed above, this indicates that other forms of insecticide treatments, either by landowners or commercial companies, may contribute to selection for *kdr* alleles, particularly at local scales, as has been shown elsewhere [45]. Vector control districts should be aware of the extent to which these largely unregulated applications may be impacting the populations they are aiming to control, and further work should be done to identify and quantify those impacts.

Importantly, all of the above analyses relied on frequencies or counts of the genetic mutations in the voltage-gated sodium channel associated with pyrethroid resistance, rather than the actual phenotype of resistance. While other mechanisms related to the outcome of resistance in this and related species have been identified [16], variation in resistance status in the *Ae. aegypti* populations studied here is highly correlated with frequencies of these mutations, as was shown in Estep et al. [18], where the authors found a Pearson correlation coefficient of 0.905 between frequencies of the IICC genotype and the permethrin resistance ratio [3].

## Conclusions

The objectives of this study were to determine the scale of spatial dependency and identify significant clusters of *kdr* genotypes in Florida *Ae. aegypti* mosquitoes, as well as develop a model explaining the variation in *kdr* genotype frequency at sampling sites throughout the state. Our findings indicate that neighboring sites can inform estimates of insecticide resistance at unknown locations, but significant clusters of resistant and susceptible genotypes can be found within a single county, meaning conclusions on resistance status should be informed by sampling at multiple locations whenever possible. While landscape variables had significant associations with the outcome of IICC frequency, the exact mechanism by which these factors modulate insecticide resistance should be investigated further.

## Supplementary information

**Additional file 1: Table S1.** Collection sites and their respective counties and count of collected and genotyped *Ae. aegypti*. **Table S2.** Coefficients and their associated level of statistical significance for the top variables in the top 20 models (without spatial lag or spatial error terms), and AIC_c_ and *R*^2^ metrics of model evaluation.

**Additional file 2: Figure S1.** Comparison of model performance as measured by *R*^2^ (left) and AIC_c_ (right) for the original, spatial lag, and spatial error versions of the original top 20 models.

**Additional file 3: Figure S2.** Variables included in the twenty models with the lowest AIC_c_ values, with *n* and the size of the rectangle denoting the number of models that included each variable, and the value of the coefficient averaged across those models. Variables that had positive associations with the response variable of IICC frequency are shown in dark gray; variables that had negative associations with the response variable of IICC frequency are shown in light gray. January LAI (A), organophosphate use (B), distance from agricultural land (C), and distance from urban or built-up land (D), were included in one model each.

## Data Availability

Data used for this research are published in Estep et al. [18].

## Abbreviations

AIC: Akaike information criterion
C: cysteine (codon 1534)
EVI: Enhanced Vegetation Index
F: phenylalanine (codon 1534)
I: isoleucine (codon 1016)
KDR: knockdown resistance
LAI: Leaf Area Index
MODIS: Moderate Resolution Imaging Spectroradiometer
SNP: single nucleotide polymorphism
ULV: ultra-low volume
V: valine (codon 1016)
