# Supplementary material for: Spatial variation in the frequency of knockdown resistance genotypes in Florida Aedes aegypti populations
Source: Parasit Vectors. 2020 May 11;13:241. doi: 10.1186/s13071-020-04112-3 (PMC7216362; doi:10.1186/s13071-020-04112-3)
Supplement: Supplementary file 1 — Additional file 1: Table S1. Collection sites and their respective counties and count of collected and genotyped Ae. aegypti. Table S2. Coefficients and their associated level of statistical significance for the top variables in the top 20 models (without spatial lag or spatial error terms), and AICc and R2 metrics of model evaluation. [file 13071_2020_4112_MOESM1_ESM.docx]

**Additional file 1: Table S1.** Collection sites and their respective counties and count of collected and genotyped *Ae. aegypti*.

| Site ID | County | n |  | Site ID | County | n |
| --- | --- | --- | --- | --- | --- | --- |
| 1 | St. Johns | 50 |  | 32 | Miami-Dade | 90 |
| 2 | St. Johns | 79 |  | 33 | Miami-Dade | 90 |
| 3 | Pinellas | 87 |  | 34 | Miami-Dade | 90 |
| 4 | Martin | 87 |  | 35 | Miami-Dade | 90 |
| 5 | Duval | 89 |  | 36 | Miami-Dade | 90 |
| 6 | Duval | 90 |  | 37 | Miami-Dade | 90 |
| 7 | Monroe | 73 |  | 38 | Miami-Dade | 88 |
| 8 | Lee | 87 |  | 39 | Miami-Dade | 87 |
| 9 | Lee | 88 |  | 40 | Miami-Dade | 83 |
| 10 | Lee | 86 |  | 41 | Miami-Dade | 90 |
| 11 | Lee | 89 |  | 42 | Miami-Dade | 88 |
| 12 | Lee | 89 |  | 43 | Miami-Dade | 88 |
| 13 | Lee | 59 |  | 44 | Miami-Dade | 88 |
| 14 | Hillsborough | 48 |  | 45 | Miami-Dade | 86 |
| 15 | Pasco | 29 |  | 46 | Manatee | 11 |
| 16 | Pasco | 25 |  | 47 | Manatee | 89 |
| 17 | Pasco | 28 |  | 48 | Manatee | 83 |
| 18 | Pasco | 29 |  | 49 | Manatee | 84 |
| 19 | Pasco | 29 |  | 50 | Manatee | 79 |
| 20 | Pasco | 29 |  | 51 | Indian River | 90 |
| 21 | Pasco | 20 |  | 52 | Orange | 85 |
| 22 | Pasco | 32 |  | 53 | Seminole | 86 |
| 23 | Pasco | 47 |  | 54 | Seminole | 85 |
| 24 | Hernando | 86 |  | 55 | Broward | 137 |
| 25 | Miami-Dade | 90 |  | 56 | Broward | 30 |
| 26 | Miami-Dade | 180 |  | 57 | Brevard | 65 |
| 27 | Miami-Dade | 180 |  | 58 | Collier | 39 |
| 28 | Miami-Dade | 180 |  | 59 | Collier | 40 |
| 29 | Miami-Dade | 88 |  | 60 | Collier | 40 |
| 30 | Miami-Dade | 89 |  | 61 | Collier | 37 |
| 31 | Miami-Dade | 89 |  | 62 | Sarasota | 61 |

**Additional file 1: Table S2.** Coefficients and their associated level of statistical significance for the top variables in the top 20 models (without spatial lag or spatial error terms), and AIC_c_ and *R*^2^ metrics of model evaluation.

| Model | Intercept | January EVI | October EVI | January LAI | July LAI | Percent tree cover | Distance from sugar-production | Distance from agricultural land | Distance from forested land | Median income | Organophosphate use | Percent non-tree vegetation cover | Pyrethroids | Distance from primary & secondary roads | Distance from urban or built-up land cover | AICc | R^2^ |
| --- | --- | --- | --- | --- | --- | --- | --- | --- | --- | --- | --- | --- | --- | --- | --- | --- | --- |
| 1 | 0.12 | 0.23* | -- | -- | 0.50* | 0.20 | -- | -- | -0.24* | -- | -- | -- | -0.47* | 0.33* | -- | -21.40 | 0.34 |
| 2 | 0.13 | 0.24* | -- | -- | 0.46* | -- | -- | -- | -0.21 | -- | -- | -- | -0.40* | 0.32* | -- | -21.06 | 0.32 |
| 3 | 0.16 | 0.32* | -- | -- | 0.47* | -- | -- | -- | -- | -- | -- | -- | -0.38* | 0.36* | -- | -20.93 | 0.30 |
| 4 | 0.15 | 0.31* | -- | -- | 0.50* | 0.18 | -- | -- | -- | -- | -- | -- | -0.44* | 0.35* | -- | -20.85 | 0.32 |
| 5 | 0.12 | -- | 0.21 | -- | 0.49* | 0.20 | -- | -- | -0.23 | -- | -- | -- | -0.47* | 0.32* | -- | -20.64 | 0.34 |
| 6 | 0.11 | -- | -- | -- | 0.46* | 0.21 | -- | -- | -0.32* | -- | -- | -- | -0.49* | 0.32* | -- | -20.54 | 0.31 |
| 7 | 0.15 | -- | 0.34* | -- | 0.46* | 0.29* | -- | -- | -- | -- | -- | -0.23 | -0.40* | 0.39* | -- | -20.51 | 0.34 |
| 8 | 0.16 | -- | 0.30* | -- | 0.45* | -- | -- | -- | -- | -- | -- | -- | -0.38* | 0.35* | -- | -20.41 | 0.30 |
| 9 | 0.15 | -- | 0.29* | -- | 0.49* | 0.19 | -- | -- | -- | -- | -- | -- | -0.44* | 0.35* | -- | -20.38 | 0.32 |
| 10 | 0.13 | -- | 0.21 | -- | 0.45* | -- | -- | -- | -0.21 | -- | -- | -- | -0.40* | 0.31* | -- | -20.26 | 0.32 |
| 11 | 0.15 | -- | 0.42* | 0.48* | -- | 0.30* | -- | -- | -- | -- | -- | -0.35* | -0.33* | 0.44* | -- | -20.25 | 0.33 |
| 12 | 0.14 | 0.27* | -- | -- | 0.44* | -- | -- | 0.18 | -0.24 | -- | -- | -- | -0.38* | 0.28* | -- | -20.23 | 0.35 |
| 13 | 0.15 | 0.32* | -- | -- | 0.45* | -- | 0.16 | -- | -- | -- | -- | -- | -0.45* | 0.37* | -- | -20.20 | 0.32 |
| 14 | 0.15 | 0.33* | -- | -- | 0.47* | 0.27* | -- | -- | -- | -- | -- | -0.19 | -0.41* | 0.39* | -- | -20.09 | 0.33 |
| 15 | 0.12 | -- | -- | -- | 0.42* | -- | -- | -- | -0.31* | -- | -- | -- | -0.41* | 0.31* | -- | -19.97 | 0.29 |
| 16 | 0.13 | 0.22 | -- | -- | 0.49* | -- | -- | -- | -0.27* | 0.14 | -- | -- | -0.42* | 0.34* | -- | -19.94 | 0.26 |
| 17 | 0.14 | 0.32* | -- | -- | 0.48* | 0.18 | 0.15 | -- | -- | -- | -- | -- | -0.51* | 0.36* | -- | -19.90 | 0.34 |
| 18 | 0.13 | 0.27* | -- | -- | 0.47* | -- | -- | -- | -0.24* | -- | 0.14 | -- | -0.35* | 0.34* | -- | -19.70 | 0.34 |
| 19 | 0.15 | 0.29* | -- | -- | 0.49* | -- | -- | -- | -- | -- | -- | -- | -0.42* | 0.36* | 0.13 | -19.51 | 0.31 |
| 20 | 0.12 | -- | -- | -- | 0.46* | -- | -- | -- | -0.36* | 0.16 | -- | -- | -0.44* | 0.33* | -- | -19.43 | 0.30 |

* p value < 0.05
