# Supplementary figures and images for: Spatial variation in the frequency of knockdown resistance genotypes in Florida Aedes aegypti populations
Source: Parasit Vectors. 2020 May 11;13:241. doi: 10.1186/s13071-020-04112-3 (PMC7216362; doi:10.1186/s13071-020-04112-3)

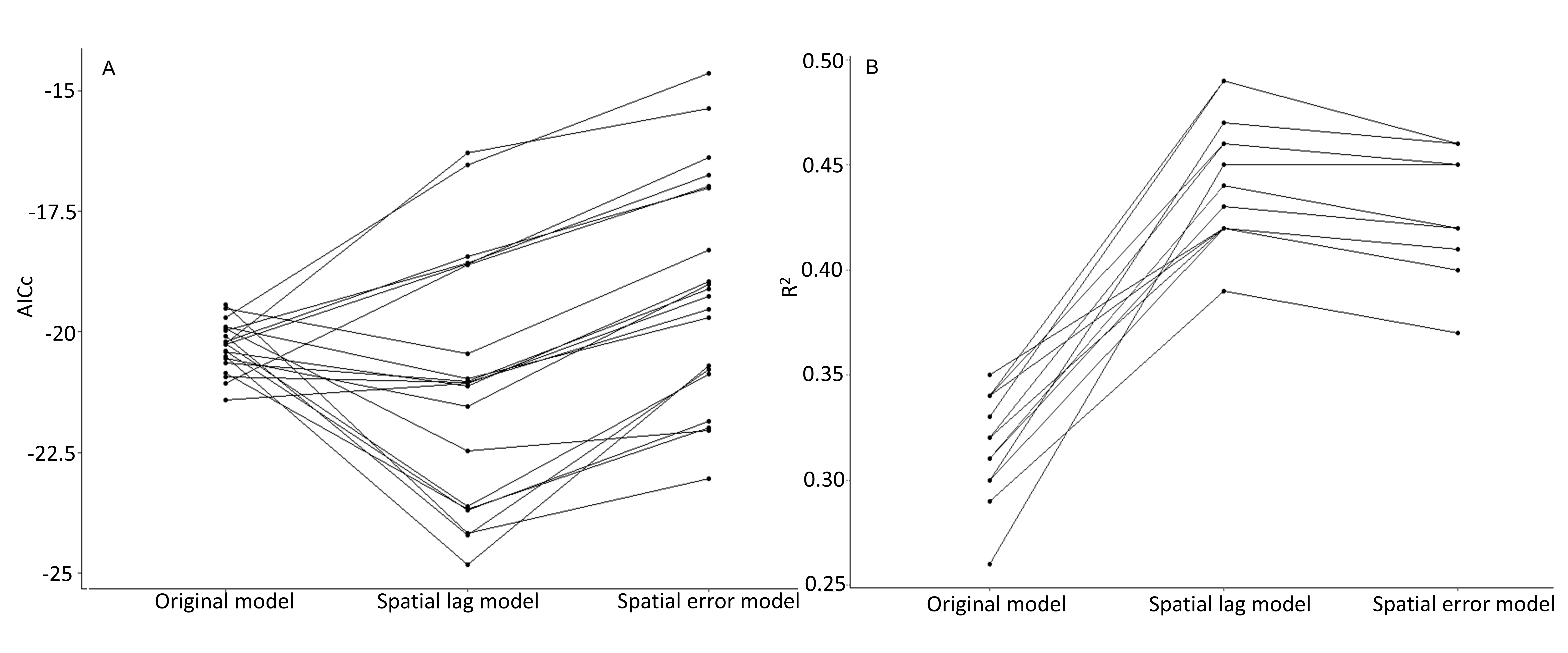

Supplement: Supplementary file 2 — Additional file 2: Figure S1. Comparison of model performance as measured by R2 (left) and AICc (right) for the original, spatial lag, and spatial error versions of the original top 20 models. [file 13071_2020_4112_MOESM2_ESM.tif]

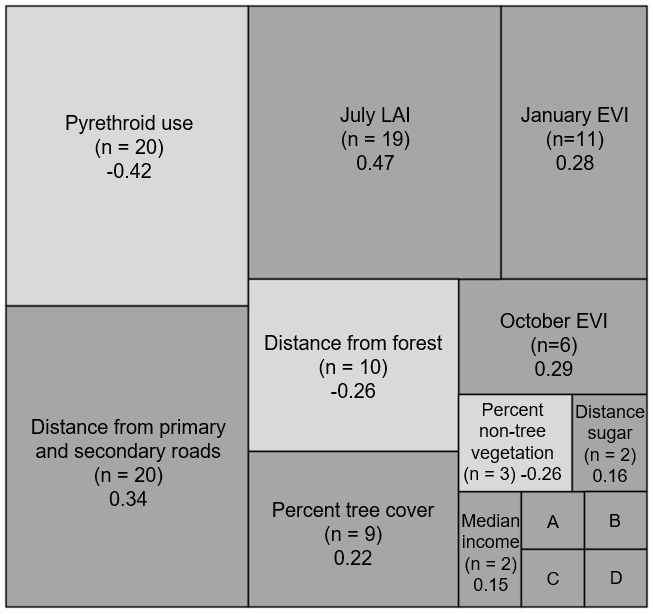

Supplement: Supplementary file 3 — Additional file 3: Figure S2. Variables included in the twenty models with the lowest AICc values, with n and the size of the rectangle denoting the number of models that included each variable, and the value of the coefficient averaged across those models. Variables that had positive associations with the response variable of IICC frequency are shown in dark gray; variables that had negative associations with the response variable of IICC frequency are shown in light gray. January LAI (A), organophosphate use (B), distance from agricultural land (C), and distance from urban or built-up land (D), were included in one model each. [file 13071_2020_4112_MOESM3_ESM.tif]
